# Beyond Molecular Determinism: State‐Convergent Polymerization as a Functional Design Principle Under Chemical Complexity

**DOI:** 10.1002/advs.76015

**Published:** 2026-06-09

**Authors:** Seonki Hong

**Affiliations:** ^1^ Department of Physics and Chemistry Daegu Gyeongbuk Institute of Science and Technology Daegu South Korea

**Keywords:** functional state convergence, melanin‐inspired polymerization, polydopamine, state‐convergent polymerization, structural heterogeneity

## Abstract

Polymeric materials are traditionally designed by prescribing molecular structures and reaction pathways. However, many functional polymers—exemplified by natural melanins and synthetic polydopamine—operate reproducibly despite persistent molecular heterogeneity and ill‐defined architectures. Here, I propose state‐convergent polymerization (SCP) as a design logic for polymeric materials formed under chemical complexity, in which polymerization is defined by convergence toward a functional material state rather than a discrete molecular structure. In SCP, polymer formation emerges from dynamically evolving pools of reactive motifs confined within environmentally bounded chemical state spaces. Crucially, these state spaces are chemically addressable through experimentally accessible variables such as pH, redox conditions, oxygen availability, and interfacial confinement, enabling multiple reaction trajectories to coexist while enforcing functional convergence. By decoupling polymer function from molecular determinism, SCP provides a materials design framework for materials operating under chemical complexity, including biointerfaces, adaptive coatings, and open‐system polymerization processes. This perspective reframes polymer synthesis from structure prescription toward state engineering, offering actionable principles for designing robust functional materials beyond molecular precision.

## Graphical abstract

This work introduces state‐convergent polymerization (SCP), a polymerization framework in which chemically diverse reaction pathways converge toward a reproducible functional polymeric state rather than a defined molecular structure. Using melanin and polydopamine as exemplars, SCP explains how robust polymer functions emerge under chemical and environmental complexity.

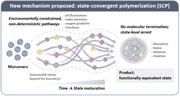

## 1 When Structure Fails: Melanin as a Challenge to Structure‐Defined Polymerization

Polymer science has long been built upon the premise that polymeric materials are defined by their molecular structures and corresponding structure–property relationships. From monomer identity and chain architecture to molecular weight distributions, structural determinism has served as the central design principle of polymer synthesis. This paradigm has enabled remarkable precision and tunability under controlled conditions [1, 2]. However, it struggles to rationalize polymeric materials whose molecular structures are intrinsically heterogeneous while their macroscopic functions remain remarkably consistent.

Natural melanins exemplify this contradiction. Despite decades of investigation, melanins resist structural definition: their monomeric composition, connectivity, and molecular weight distributions vary across species, tissues, environmental conditions, and even synthesis or isolation histories [3, 4]. Unlike most synthetic polymers, melanins cannot be described by a well‐defined repeat unit, monomer sequence, or discrete molecular architecture. Instead, they are more accurately understood as chemically heterogeneous ensembles rather than single macromolecular entities [4, 5, 6].

This intrinsic heterogeneity places melanin outside the conceptual boundaries of structure‐defined polymerization. Classical polymer synthesis relies on fixed monomer identities, predictable connectivity, and controllable growth pathways, allowing polymeric materials to be rationally designed through molecular structure. Melanin formation, in contrast, proceeds through complex oxidative reactions involving catechol‐ and indole‐derived species whose chemical identities continuously evolve during the process [3, 4, 5]. The resulting polymeric matter defies reduction to a discrete molecular structure, rendering many standard descriptors of polymer chemistry—such as degree of polymerization, chain architecture, or molecular weight distribution—largely inapplicable.

Importantly, this lack of structural definition is not a consequence of insufficient analytical resolution or incomplete mechanistic understanding. Extensive spectroscopic, chromatographic, and computational efforts have repeatedly demonstrated that no consensus molecular structure emerges. Rather, structural indeterminacy is a fundamental feature of melanin formation itself [3, 5]. Yet paradoxically, this molecular ambiguity does not propagate into functional ambiguity. Across biological contexts and preparation conditions, melanins consistently exhibit a characteristic set of material functions, including broadband optical absorption, redox activity, radical scavenging capability, and strong affinity for metal ions [3, 5].

The robustness of these functionalities suggests that melanin formation is governed by selection at the level of material performance rather than molecular structure. In biological systems, melanins are not required to adopt a specific architecture; they are required to perform reliably under fluctuating and often harsh environmental conditions. As long as the emergent polymeric material satisfies these functional demands, variability at the molecular level appears to be tolerated, or even advantageous.

The coexistence of structural indeterminacy and functional reproducibility challenges the structure‐defined framework that dominates synthetic polymer chemistry. Melanin formation suggests that polymer synthesis need not converge toward a specific molecular architecture to achieve reliable function. Instead, it points toward a different organizing principle—one in which chemically heterogeneous and environmentally responsive pathways nonetheless converge toward a stable functional outcome. Addressing this discrepancy requires moving beyond structure as the primary product of polymerization and toward a framework in which polymerization is understood as the emergence of a functional state.

## 2 Defining State‐Convergent Polymerization

**FIGURE 1:**
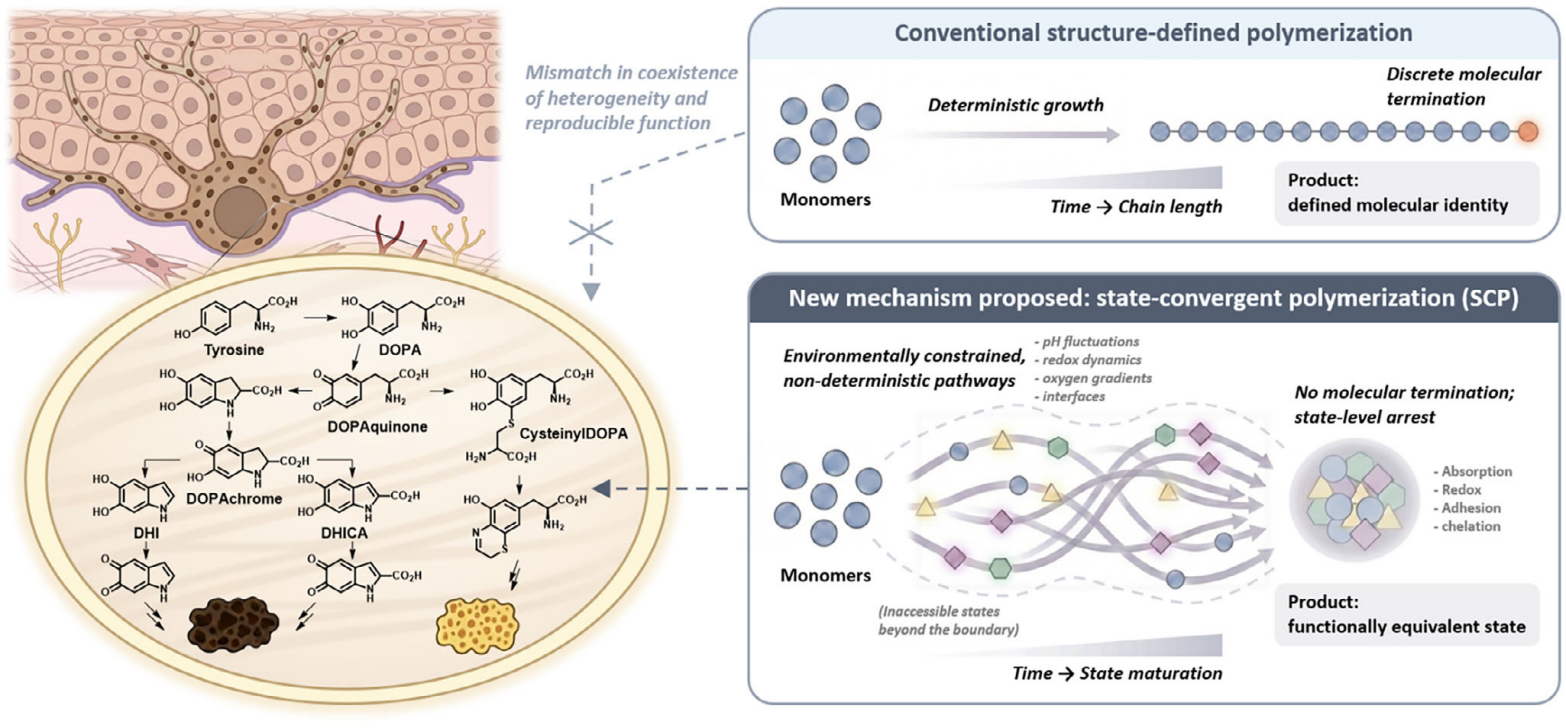
Structure‐defined polymerization versus state‐convergent polymerization (SCP) inspired by melanogenesis. In structure‐defined polymerization (top right), polymer growth proceeds deterministically under narrowly defined reaction conditions, converging toward a product with a well‐defined molecular identity through discrete molecular termination. In contrast, SCP (bottom right) operates within an environmentally and process‐constrained chemical state space, where dynamically evolving reactive motifs explore multiple non‐deterministic pathways. Although individual molecular trajectories remain heterogeneous, polymerization converges through state‐level arrest toward a functionally equivalent material state rather than a specific molecular architecture. The left illustrates melanogenesis as a natural exemplar of SCP, highlighting how chemical diversification of monomeric precursors through oxidation and coupling reactions gives rise to reproducible material function despite persistent molecular heterogeneity.

The paradox revealed by melanin formation—the coexistence of intrinsic structural heterogeneity with robust functional reproducibility—calls for a polymerization framework that does not assume convergence toward a well‐defined molecular structure. Here, I argue that polymerization can instead be deliberately directed toward convergence at the level of function, even when the underlying molecular architectures remain indeterminate. This behavior is captured by the concept of state‐convergent polymerization (SCP) (Figure 1). In SCP, the outcome of polymerization is defined not by a specific molecular architecture, but by the emergence of a reproducible functional polymeric state. Polymer formation proceeds through a dynamically evolving pool of reactive motifs whose chemical identities continuously transform during the process, making molecular heterogeneity an intrinsic characteristic rather than a synthetic imperfection [5, 6, 7, 8].

The classification of SCP products as polymers challenges the assumption that polymers must be defined as discrete molecular entities. Although SCP products resist reduction to well‐defined macromolecules, they exhibit hallmark polymeric behaviors, including network formation, non‐volatility, processability, and functional continuity [3]. SCP products such as melanin are therefore polymers whose defining characteristics emerge at the level of the material state rather than from a specific molecular identity.

A defining characteristic of SCP lies in how polymerization terminates and converges. In SCP, termination is not defined by a discrete molecular event such as chain termination or catalyst deactivation. Instead, polymerization proceeds until the system reaches a state‐level arrest, beyond which further chemical evolution no longer alters the functional identity of the material. This arrest emerges through a combination of processes, including depletion of reactive motifs, aggregation‐induced immobilization, saturation of interfacial sites, and equilibration among redox‐active species [9, 10, 11], which arise as emergent consequences of the bounded chemical state space imposed by environmental and process constraints.

Reaction conditions such as pH, redox potential, oxygen availability, and interfacial constraints are known to strongly influence melanogenesis and related processes [11, 12, 13, 14]. Within the SCP framework, these factors do not merely modulate reaction kinetics; they define which chemical states and reaction pathways are accessible throughout polymer formation. Polymerization therefore occurs within a bounded process window, where multiple chemically distinct polymerization trajectories can evolve in parallel yet remain confined to the same functional outcome. Within this window, continued molecular heterogeneity does not propagate into divergent material behavior. Instead, diverse polymerization pathways converge toward an equivalent functional state, providing a mechanistic basis for functional reproducibility in the absence of molecular determinism. As a result, polymer formation exhibits self‐limiting convergence, ceasing upon attainment of a stable functional polymeric state rather than a specific molecular endpoint. Importantly, such convergence does not necessarily reflect attainment of a unique global thermodynamic minimum, nor merely accidental kinetic trapping within a heterogeneous mixture. Rather, SCP proposes that chemically accessible trajectories become progressively restricted within an environmentally bounded state space. In this way, unlike conventional kinetic traps, which often produce path‐dependent or irreproducible arrested states [15], SCP preserves reproducible functional outcomes despite underlying chemical heterogeneity.

Crucially, SCP tolerates not only molecular heterogeneity but also compositional evolution of the reactive motif pool [6, 16]. Depending on the extent of chemical reprogramming, such evolution can either preserve convergence toward the same functional basin or shift the bounded chemical state space toward a new, redefined basin while maintaining state‐level convergence. This dual mode of convergence highlights that SCP is not restricted to a single chemical composition or reaction network, but instead accommodates adaptive reconfiguration of the reactive landscape as long as polymerization remains confined within a bounded and chemically addressable state space.

Importantly, this state‐based logic is already encoded within melanogenesis itself, where variations in precursor availability reshape the reactive landscape without abolishing functional convergence. Within the same melanogenic framework, pheomelanin formation provides a particularly clear illustration of basin‐level reorganization under SCP. During melanogenesis, cysteine incorporation diverts catechol oxidation pathways toward sulfur‐containing benzothiazine and benzothiazole motifs, fundamentally reshaping the reactive motif pool [17]. This chemical reprogramming does not merely perturb reaction kinetics but redefines the bounded chemical state space itself. As a consequence, polymerization trajectories no longer converge toward the eumelanin basin but are redirected toward a distinct pheomelanin basin, characterized by a different ensemble of chemical motifs and functional signatures.

Practical criteria for identifying when polymer formation operates in a state‐convergent regime are summarized in Table 1. By decoupling polymer function from molecular structure, SCP reframes polymerization as a process of functional state emergence rather than molecular structure definition, providing a conceptual foundation for understanding polymeric materials that operate reliably under chemically heterogeneous and dynamically fluctuating conditions. Importantly, SCP does not represent a relaxation of synthetic rigor, but a reallocation of control from molecular architecture to environmental and process variables. Polymerization is thus best understood as a state transition toward a functionally convergent polymeric state, through which systems previously regarded as ill‐defined or poorly controlled can instead be recognized as valid—and deliberately accessible—instances of SCP.

**TABLE 1:** Key conceptual differences between conventional polymer synthesis and state‐convergent polymerization (SCP).

Category	Conventional structure‐defined polymerization	State‐convergent polymerization (SCP)
Primary definition of polymerization	Formation of polymer molecules	Emergence of a functional polymeric state
Identity of the product	Discrete macromolecules	Material state defined by converged functionality
Fundamental building unit	Monomer	Evolving reactive motif pool
Monomer identity	Fixed and predefined	Dynamic, chemically evolving
Reaction scheme	Specified a priori	Emergent and adaptive
Reaction pathways	Limited, predefined	Multiple, environmentally shaped
Role of environment	Modulates kinetics, distributions, and microstructure within a fixed reaction scheme	Defines accessible chemical states and reaction networks
Growth description	Chain‐growth or step‐growth	Progressive assembly
Reaction domain	Bulk solution	Process‐ and interface‐confined
Structural determinism	High	Low (emergent structure)
Functional determinism	Derived from molecular structure	Primary design target
Termination concept	Discrete molecular termination events	State‐level arrest via self‐limiting convergence
Mechanisms of growth arrest	Radical termination, chain transfer, catalyst deactivation	Reactive motif depletion, aggregation‐induced arrest, interfacial saturation, redox equilibration
Time dependence	Correlates with molecular weight and conversion	Correlates with development of functional state
Reproducibility criterion	Molecular identity and distribution	Functional consistency
Interpretation of variability	Synthetic imperfection or noise	Adaptive and tolerated heterogeneity
Design philosophy	Structure‐first	Function‐first (state‐driven)

**FIGURE 2:**
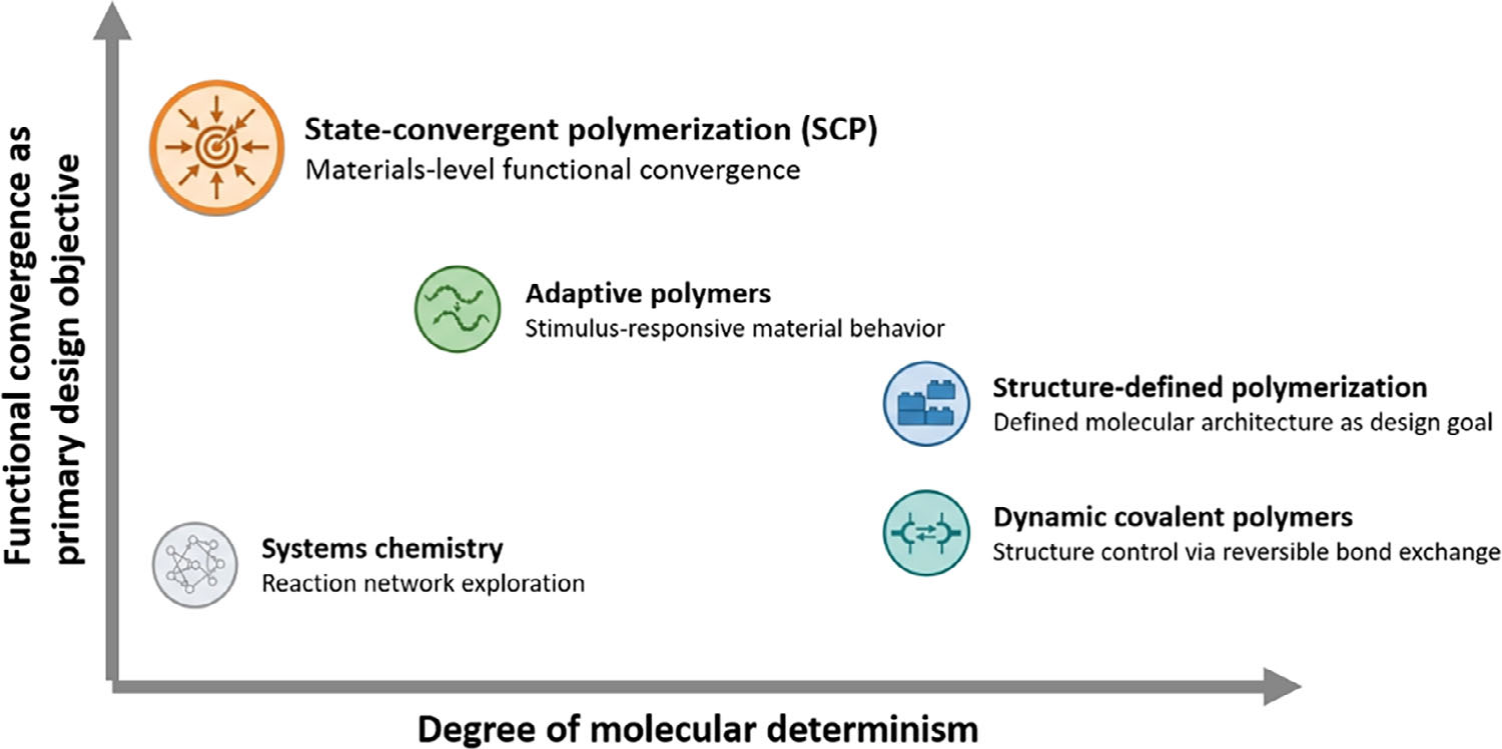
Conceptual positioning of state‐convergent polymerization (SCP) among adjacent polymerization paradigms. The horizontal axis represents the degree of molecular determinism assumed during polymer formation, ranging from low to high reliance on predefined molecular architecture. The vertical axis represents the extent to which functional convergence is treated as the primary design objective. SCP occupies a conceptual space characterized by low molecular determinism and explicit targeting of materials‐level functional convergence. The positions are schematic and intended to illustrate differences in design orientation rather than strict categorical boundaries.

To further clarify the conceptual scope of SCP, it is useful to distinguish it from several adjacent and partially overlapping research paradigms beyond conventional structure‐defined polymerization. Emerging fields such as systems chemistry, adaptive polymers, and dynamic covalent polymers share with SCP an interest in dynamic behavior and environmentally responsive chemical processes, yet they differ in their primary design orientation (Table 2). Systems chemistry, for instance, centers on the exploration and control of emergent behaviors in interacting chemical networks, often under out‐of‐equilibrium or chemically fueled conditions, and primarily seeks to understand network dynamics and organization rather than to direct polymer formation toward a reproducible functional material state [18]. Non‐equilibrium and dissipative materials likewise operate outside thermodynamic equilibrium and rely on continuous energy input to sustain dynamic states or spatiotemporal patterns; although they frequently involve chemically complex reaction networks, they do not generally pursue state‐level functional convergence across heterogeneous polymerization trajectories [19]. Beyond network‐level dynamics, adaptive polymers emphasize environmental responsiveness and functional variability at the materials level, but this responsiveness typically arises after material formation rather than through the deliberate convergence of diverse polymerization pathways during synthesis [20, 21]. Dynamic covalent polymer systems similarly enable reversible structural reorganization, error correction, and reprocessability through bond exchange mechanisms, yet their design logic remains fundamentally structure‐centric, as control is exerted within defined molecular or network architectures [22, 23, 24]. As summarized in Figure 2, SCP occupies a distinct conceptual space in which polymer formation is intentionally guided by state‐level convergence rather than structural definition.

**TABLE 2:** Conceptual overlaps and distinctions between state‐convergent polymerization (SCP) and adjacent polymer/material paradigms.

Paradigm	Shared features with SCP	Key conceptual distinction from SCP
Systems chemistry	Dynamic reaction networks; emergent behavior; non‐equilibrium organization	Primarily seeks to understand or control network dynamics rather than directing polymer formation toward reproducible functional material states
Adaptive polymers	Environmental responsiveness and functional adaptability	Responsiveness typically emerges after material formation rather than through convergence of heterogeneous polymerization pathways
Dynamic covalent polymers	Structural adaptability and reversible reconfiguration	Design logic remains fundamentally structure‐centric and dependent on predefined molecular or network architectures
Non‐equilibrium / dissipative materials	Dynamic states sustained under non‐equilibrium conditions	Does not generally pursue convergence toward reproducible functional material states across heterogeneous reaction trajectories

## 3 Polydopamine as a Synthetic Realization of State‐Convergent Polymerization

Beyond melanogenesis, related state‐convergent characteristics may also emerge in other naturally occurring chemically heterogeneous polymerization systems, including lignification and humic substance formation [25, 26]. These systems likewise involve distributed oxidative coupling reactions, dynamically evolving reactive ensembles, and environmentally constrained reaction networks that generate structurally heterogeneous yet functionally persistent materials. However, such examples remain largely confined to biological or environmental contexts. At present, experimentally accessible synthetic realizations of SCP remain extremely limited, with polydopamine representing perhaps the clearest and most tractable system currently available.

Since its introduction as a universal surface coating [27], polydopamine has frequently been described as an ill‐defined polymer produced through poorly controlled oxidative polymerization of dopamine [9, 28, 29]. When revisited through the SCP framework, however, these same characteristics—structural heterogeneity, environmental sensitivity, and functional robustness—emerge not as deficiencies but as defining features of the polymerization process.

**FIGURE 3:**
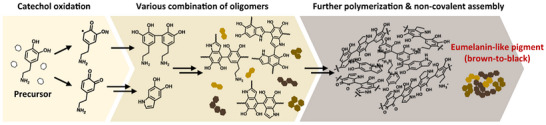
Progressive assembly during polydopamine formation reported in previous studies. Schematic representation of progressive assembly observed during polydopamine formation, in which chemically diverse intermediates generated during dopamine oxidation continuously participate in hierarchical assembly while progressively acquiring new functional characteristics. Reproduced under the terms of the Creative Commons Attribution 4.0 International License (CC‐BY 4.0) [30], Copyright 2023, The Authors, Published by Springer Nature.

At the chemical level, this reinterpretation necessitates moving beyond monomer‐centric descriptions of polymer growth. Similar to melanin formation, polydopamine synthesis does not proceed from a fixed monomer identity toward a deterministic molecular architecture. Although dopamine is introduced as a molecular precursor, its chemical identity rapidly diversifies through oxidation, cyclization, rearrangement, and intermolecular coupling reactions. These species participate collectively in intermolecular assembly, a process previously described as progressive assembly (Figure 3) [30, 31]. Importantly, this assembly process is not sequentially separable from ongoing covalent chemical transformations, but dynamically coupled with them throughout material formation, continuously reshaping accessible reaction pathways and intermolecular interactions. As in melanin formation, no single reaction pathway dominates polydopamine growth, and the process cannot be reduced to classical chain‐growth or step‐growth polymerization mechanisms. Within the SCP framework, such behavior is reinterpreted not merely as a descriptive growth mode, but as the manifestation of a dynamically evolving reactive‐motif pool, comprising catechol, quinone, indole‐like, and oligomeric species whose relative populations continuously evolve and converge.

A defining feature of polydopamine formation is its strong process confinement. Polymerization occurs only within a narrow set of environmental conditions, typically requiring alkaline pH, dissolved oxygen, and the presence of a substrate interface [32, 33, 34, 35]. These conditions do not merely modulate reaction rates; they actively shape the reactive landscape by governing oxidation states, solubility, aggregation, and surface adsorption. Polymer formation is therefore inseparable from the coating process itself, and growth is inherently interfacial and self‐limiting rather than freely propagating in bulk solution. In SCP terms, these constraints define the bounded chemical state space within which polydopamine formation can proceed and ultimately converge.

Despite this chemical and structural complexity, polydopamine exhibits striking functional convergence. Across substrates, batch conditions, and experimental implementations, polydopamine coatings reproducibly display strong adhesion, broadband optical absorption, redox activity, metal chelation, and chemical reactivity toward secondary functionalization [32, 36, 37, 38]. These functionalities persist even as molecular composition and nanoscale organization vary, indicating that polymerization converges toward a stable functional state rather than a defined molecular product. Within the SCP framework, polydopamine thus represents a synthetic realization of a polymerization logic long employed by nature, in which chemical diversity is tolerated while convergence is enforced at the level of material function.

Crucially, the robustness of this convergence extends to deliberate compositional expansion of the reactive motif pool. In one‐pot polydopamine coating strategies, diverse co‐reactants—including thiols, amines, polymers, and metal ions—are intentionally introduced during dopamine oxidation [32]. These additives profoundly alter the local composition of reactive species and introduce new coupling possibilities, effectively rewiring portions of the reaction network. However, they do not fundamentally redefine the accessible chemical state space of polydopamine formation. As a result, polymerization trajectories remain confined within the original polydopamine basin, and convergence toward the same functional polydopamine state is preserved despite substantial compositional perturbations. Hallmark properties such as adhesion, redox activity, and interfacial robustness are retained, even as the underlying molecular ensemble becomes more chemically diverse.

Recognizing polydopamine as an SCP system carries predictive and design implications, explaining why attempts to impose strict molecular control often fail, why coating thickness and functionality saturate despite continued reaction time, and why reproducibility is achieved at the level of performance rather than composition. Polydopamine thus serves as a proof‐of‐concept that SCP can be deliberately accessed in synthetic systems.

## 4 Why We Must Learn from Nature: The Rationale for State‐Convergent Polymerization

The motivation to adopt SCP extends beyond a conceptual reconsideration of polymer definitions. It arises from a fundamental mismatch between the assumptions underlying structure‐defined polymer synthesis and the increasingly diverse formation regimes of emerging functional materials, which now span both biologically regulated environments, such as in situ formation at biointerfaces, and synthetic process environments, including continuous‐flow systems and open, ambient‐condition manufacturing, where materials are formed and operated under inherently non‐equilibrium conditions.

Biological environments rarely permit precise molecular control [39, 40], as fluctuations in pH, redox potential, reactant availability, and spatial confinement are intrinsic rather than exceptional. Nevertheless, biological polymeric materials must perform reliably despite such variability. Melanin formation exemplifies how nature resolves this constraint: rather than enforcing convergence toward a specific molecular architecture, polymerization proceeds through chemically heterogeneous pathways that nonetheless converge toward a stable functional state. This natural strategy highlights a polymerization logic that is inherently compatible with materials platforms operating under unavoidable environmental complexity.

As materials synthesis increasingly moves beyond idealized reaction vessels toward open systems and ambient conditions—driven, for example, by sustainable and green manufacturing paradigms—the same constraints begin to apply to synthetic materials. Under such regimes, strategies that rely on strict monomer definition, deterministic reaction pathways, and molecular‐level reproducibility become difficult to implement and, in some cases, counterproductive. SCP offers a framework that is inherently compatible with these environments by treating environmental variability as a design input rather than a source of error.

**FIGURE 4:**
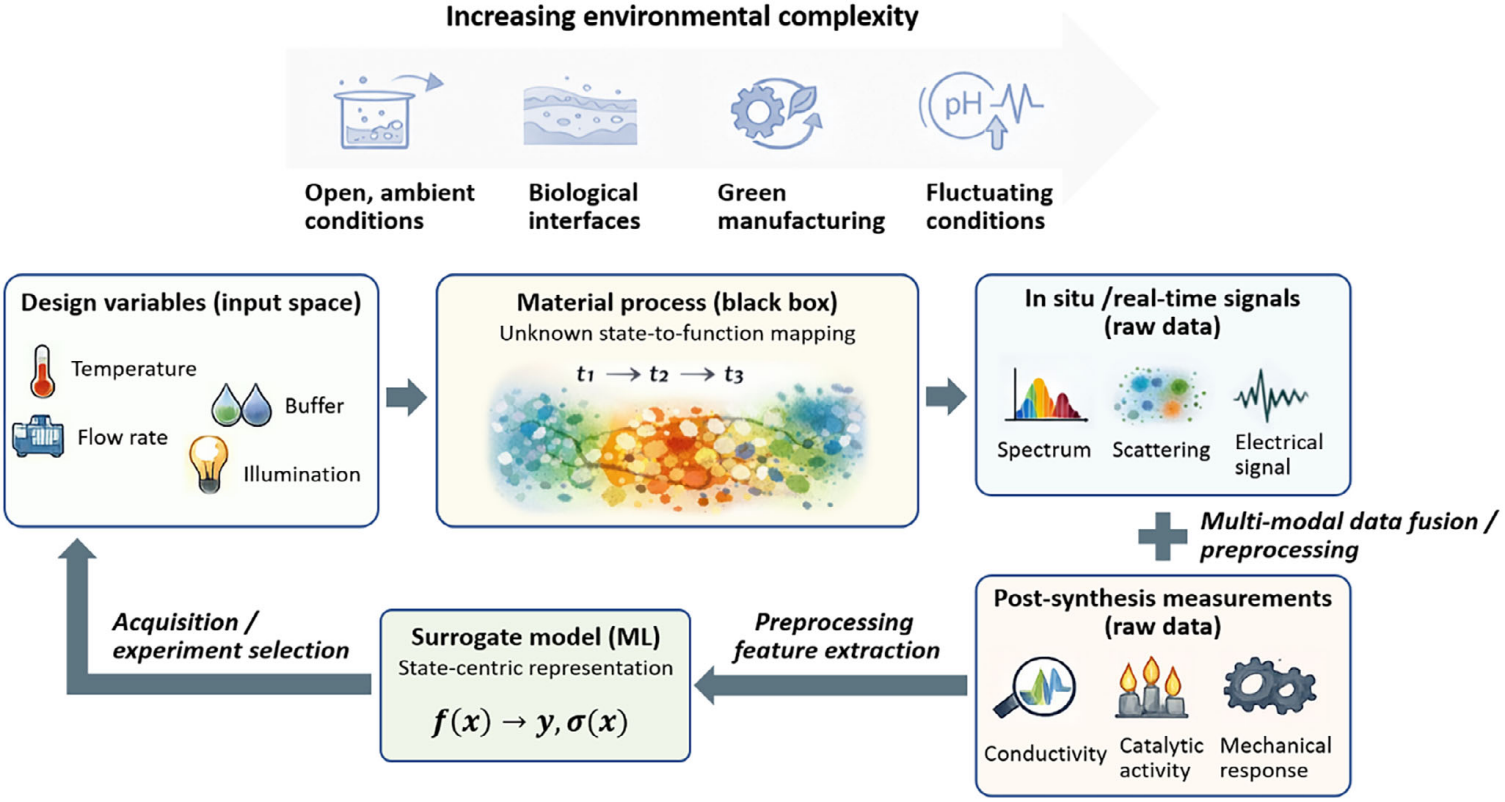
SCP‐based, state‐centric closed‐loop materials design framework. Schematic illustration of an SCP‐based, data‐driven materials design workflow in which experimentally addressable design variables are iteratively optimized through a closed‐loop learning process. A material process operating under open and non‐equilibrium conditions is treated as a black box with an unknown state‐to‐function mapping. Multi‐modal experimental data, collected via in situ and real‐time measurements as well as post‐synthesis characterization, are integrated through data fusion and preprocessing and used to train a surrogate machine‐learning model with a state‐centric representation. The model provides predictive mean and uncertainty estimates, which guide adaptive experiment selection and enable iterative optimization of functional performance. In practice, the specific experimental, analytical, and learning components may be instantiated in multiple modules and hierarchical loops; the schematic highlights the conceptual organization rather than a specific implementation.

Importantly, this shift coincides with a broader transformation in contemporary materials research, in which data‐driven and AI‐assisted approaches are increasingly used to guide materials discovery and optimization [41, 42, 43]. Such approaches remain largely dependent on well‐defined structural descriptors, implicitly assuming that molecular identity can be resolved and controlled. However, many emerging functional materials—particularly those formed in open, non‐equilibrium, and in situ environments—do not admit such structure‐centric representations [44]. In these regimes, materials design must be reframed from stable molecular descriptors toward experimentally addressable state variables and multi‐modal functional observables, which can be learned, predicted, and optimized directly from data. SCP directly addresses this growing mismatch by enabling a state‐centric, closed‐loop learning framework that integrates in situ and post‐synthesis experimental measurements and supports adaptive optimization of material function under non‐equilibrium conditions (Figure 4). These observables may include spectral fingerprints (e.g., UV–vis, Raman, and IR responses), electrochemical and redox profiles, aggregation and scattering signatures, interfacial energies and adhesion characteristics, deposition kinetics, and other in situ process measurements. Importantly, such observables are inherently time‐dependent and history‐dependent, collectively encoding dynamically evolving trajectories through environmentally bounded chemical state spaces rather than fixed molecular identities.

Learning from nature therefore does not imply mimicking specific molecular structures, but adopting a polymerization logic optimized for robustness under complexity. Adopting SCP expands the design space of polymer synthesis by allowing multiple chemically distinct polymerization trajectories to converge toward equivalent functional states. By relinquishing the requirement for molecular determinism, SCP enables tolerance to imperfect inputs, environmental fluctuations, and process variability, while preserving reproducibility at the level that ultimately matters—material function. In this sense, SCP represents not an alternative curiosity, but a necessary complement to structure‐defined synthesis as polymer science confronts increasingly complex material challenges.

## 5 Design Principles of State‐Convergent Polymerization

**FIGURE 5:**
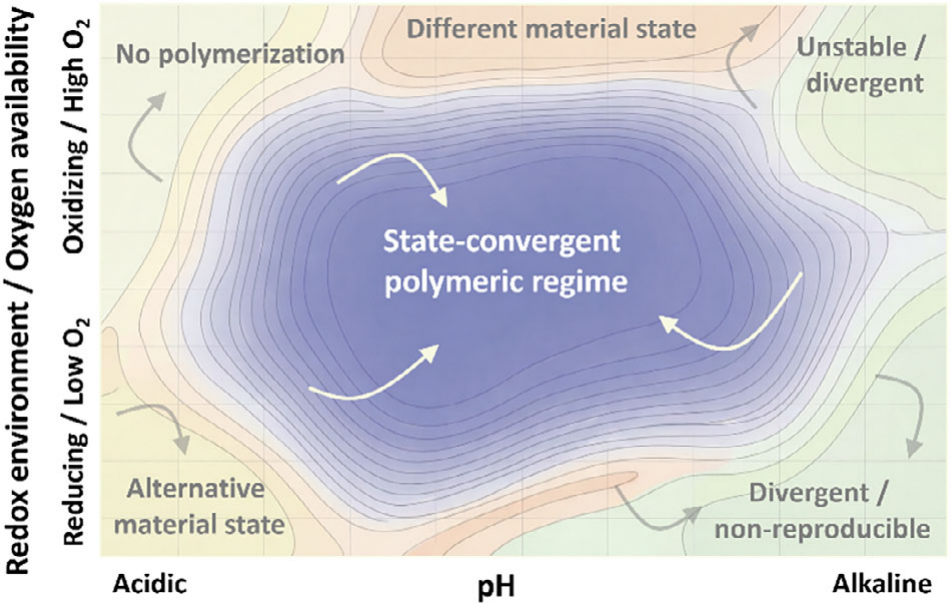
Process‐state map of state‐convergent polymerization (SCP). Within a bounded environmental window (blue region), distinct reaction trajectories originating from different initial states converge toward a functionally equivalent SCP state despite persistent molecular heterogeneity. Here, time reflects state maturation rather than progressive molecular definition, and convergence arises through state‐level arrest rather than discrete molecular termination. Outside this regime, polymerization may fail, diverge into unstable or non‐reproducible outcomes, or yield alternative material states with distinct functional identities. The schematic illustrates that SCP does not result from unrestricted chemical complexity, but from convergence within a constrained environmental state space that privileges polymeric function over molecular structure. The environmental axes shown (e.g., pH and redox conditions) are representative; depending on the system, SCP state spaces may be defined by other environmental, interfacial, or process variables.

As polymer synthesis increasingly operates under constraints similar to those shaping natural polymeric materials, these principles provide a framework for translating nature's state‐based logic into deliberate synthetic design. Unlike structure‐defined polymerization, where design is encoded in monomer identity and reaction pathways, SCP is designed by shaping the chemical state space within which polymerization unfolds. Control is therefore exercised not over molecular architecture, but over the environmental and process variables that constrain and guide state convergence (Figure 5).

A first design principle of SCP is the regulation of the evolving reactive motif pool. Instead of fixing monomer structure, SCP systems modulate the composition and evolution of reactive species through chemically accessible variables such as pH, redox potential, oxygen availability, and precursor ratios. These parameters determine which motifs are accessible, how long they persist, and how they interact. Design thus focuses on shaping the reactive landscape rather than prescribing specific reaction pathways. Beyond environmental regulation, SCP can also be engineered through deliberate expansion of the reactive motif pool without sacrificing state convergence. The aforementioned one‐pot polydopamine coating strategies exemplify this design principle, in which diverse co‐reactants are introduced during polymerization. Although these additives profoundly alter the local composition of reactive species and introduce new coupling possibilities, the overall reaction network remains confined within the accessible chemical state space and converges toward the same functional basin, while enabling modular functional diversification.

A second principle is spatial and process confinement, which enforces convergence by physically bounding polymer formation. SCP relies on restricting polymerization within a defined process window, often through interfacial constraints, limited solubility, aggregation‐induced arrest, or spatial compartmentalization. These constraints prevent unbounded chemical exploration and promote self‐limiting convergence toward a stable functional state. Spatial confinement provides a particularly powerful handle for enforcing state convergence. By physically restricting reaction volumes—such as within polymersomes, nanocavities, or compartmentalized polymer matrices—the accessible chemical state space can be sharply bounded in both space and time. Recent studies on confined melanin and polydopamine formation demonstrate that such spatial constraints profoundly influence oxidation pathways, reactive motif lifetimes, and aggregation dynamics, while still enabling convergence toward coherent functional states [30, 45, 46, 47, 48]. Importantly, confinement does not impose molecular determinism; rather, it limits the exploration of chemical space, accelerating state‐level arrest and enhancing reproducibility under chemically complex conditions.

Temporal control constitutes a third design principle of SCP. In contrast to conventional polymerization, where time correlates with molecular weight or degree of polymerization, time in SCP reflects the degree of state maturation. Deposition time, exposure history, and environmental fluctuations determine how extensively the system explores its accessible chemical state space before convergence. As a result, polymerization is operationally understood as progressive assembly rather than discrete molecular growth. Functional saturation, rather than molecular conversion, serves as the practical indicator of polymerization completion.

A fourth principle is the prioritization of functional validation over structural minimization. SCP evaluates success through the emergence and saturation of material functions—such as adhesion, redox activity, optical response, or interfacial stability—rather than through molecular descriptors alone. Structural characterization remains important, but its role is reframed: heterogeneity becomes a variable to be tolerated or leveraged, rather than eliminated. Functional reproducibility, rather than molecular uniformity, serves as the primary benchmark of synthetic success.

Together, these principles define SCP as a deliberate and engineerable polymerization strategy in which relinquishing molecular determinism enables control at scales more relevant to material performance. By designing chemical state spaces—through compositional, spatial, and temporal constraints—rather than molecular architectures, SCP provides a practical route to polymeric materials that operate reliably under conditions of chemical complexity.

## 6 Conclusion

State‐convergent polymerization (SCP) calls into question a long‐standing assumption in polymer science—that polymer identity must be anchored in a well‐defined molecular structure. Using natural melanins and their synthetic analogue, polydopamine, as exemplars, this perspective illustrates how polymeric materials can reliably acquire characteristic functions even when their molecular architectures remain heterogeneous and unresolved.

SCP does not seek to replace structure‐defined polymerization, nor does it diminish the value of molecular precision. Rather, it delineates a complementary regime of polymer formation in which functional reproducibility arises from convergence within chemically bounded state spaces, rather than from deterministic molecular growth. By framing polymerization as a state‐level transition rather than a molecular endpoint, SCP provides a practical basis for interpreting and designing polymeric materials whose performance cannot be rationalized through molecular descriptors alone. This perspective enables functional polymers to be evaluated and compared on the basis of converged material behavior, offering a route toward synthesis strategies that emphasize robustness and adaptability under chemically complex conditions.

From a practical standpoint, SCP shifts the focus of polymer research away from prescribing molecular architectures and toward identifying which chemically controllable variables reproducibly select a desired functional state. Rather than attempting to eliminate molecular heterogeneity, this approach encourages researchers to identify and tune environmental parameters—such as redox potential, pH, confinement, and interfacial dynamics—that bound the accessible reaction network and enforce functional convergence. In doing so, SCP provides tolerance to imperfect inputs, fluctuating conditions, and open‐system operation by harnessing, rather than suppressing, chemical heterogeneity within chemically addressable state spaces.

By formalizing state convergence as a design target, this work invites polymer and materials researchers to reconsider how functional reproducibility can be achieved when molecular determinism is neither attainable nor necessary.

## Author Contributions

**Seonki Hong**: conceptualization, investigation, writing – original draft, writing – review and editing, project administration, visualization.

## Conflicts of Interest

The author declares no conflicts of interest.

## Data Availability

Data sharing not applicable to this article as no datasets were generated or analyzed during the current study.
